# Experts’ and Novices’ Perception of Ignorance and Knowledge in Different Research Disciplines and Its Relation to Belief in Certainty of Knowledge

**DOI:** 10.3389/fpsyg.2017.00377

**Published:** 2017-03-17

**Authors:** Isabelle Hansson, Sandra Buratti, Carl Martin Allwood

**Affiliations:** Department of Psychology, University of Gothenburg, GothenburgSweden

**Keywords:** ignorance, knowledge assessments, experts, novices, belief in certainty of knowledge

## Abstract

Assessments of the extent of knowledge in a domain can be important since non-identified lack of knowledge may lead to decisions that do not consider the effect of relevant factors. Two studies examined experts’ and novices’ perception of their own ignorance and knowledge *out of everything there is to know* within their own and other disciplines and their assessments of their discipline’s, and other disciplines’ knowledge of all there is to know in each discipline. In total 380 experts and 401 students from the disciplines of history, medicine, physics, and psychology participated. The results for ignorance and knowledge assessments of one’s own knowledge were similar. Novices reported more ignorance and less knowledge in their own discipline than experts, but no differences were found in the assessments of how much is known in each discipline. General belief in certainty of knowledge was associated with the knowledge assessments and level of expertise. Finally, disciplinary differences were found both for the knowledge assessments and for belief in certainty of knowledge. Historians and physicists assessed that less was known in their own discipline out of all there is to know (approximately 40%), compared to the medics (about 50%). Historians believed least in certainty of knowledge and physicists most. Our results have practical implications for higher educational teaching and interdisciplinary collaboration.

## Introduction

In order for people to make good judgments and decisions it is often important for them to consider the extent of their own and others’ knowledge about the deliberated issue. Generally speaking, lack of knowledge implies uncertainty in judgments and a risk that the decision made is wrong (Edwards and Tversky, 1967), for example because the decision does not consider the effect of all relevant factors. Previous research has studied people’s knowledge assessments in fairly limited contexts (e.g., the extent of their knowledge in the area covered by a university test to be taken), but in real life any factors that influence events may be relevant to consider, irrespective of if these factors have been previously identified or not. It is therefore important to improve our understanding of broader knowledge assessments relating to all there is to know in an area.

To lack relevant knowledge without realizing it can have disastrous consequences. Consider, for example, the underestimation of the risk of a nuclear accident when constructing the Fukushima power plant in Japan (e.g., Ravetz, 1993), the risk of doctors making premature decisions on the basis of too limited information (Groopman, 2007), the risk of juries prematurely reaching a “guilty” (or “non-guilty”) outcome, or newspapers prematurely deciding to publish a news-story, etc. Careful assessments of the extent of one’s own and other’s knowledge may help hinder such events. Furthermore, given humans’ general tendency to look for affirmative examples (Nickerson, 1998) it can be important to consider *lack of knowledge* within a certain area, although this may be less common to do. In general, researchers’ knowledge tends to be trusted and research tends to play an ever larger role in the planning and development processes of society. Therefore researchers’ assessments of lack of knowledge may be especially pertinent to understand better. An example where this may be relevant is decisions about allocation of research funding.

The present study explored how academic experts and novices differ in their assessment of *their own ignorance* (Study 1) and of *their own knowledge* (Study 2) in relation to everything there is to know in a number of subareas in their own discipline, and these ratings were compared to ratings of their own knowledge within a number of subareas in four different scientific disciplines. Furthermore, people often rely on the knowledge of others in society and experts and novices may hold different conceptions about their own knowledge within a field and of the state of knowledge in the field as such. Therefore we also investigated participants’ assessments of how much is known *within the same four disciplines as such* in relation to everything there is to know. Finally, in order to learn more about how knowledge assessments relate to the assessor’s thinking style, we also investigated the relationship between the participants’ epistemological belief in certainty of knowledge and the knowledge assessments made by the same university experts and novices.

Research has investigated people’s knowledge assessments in different contexts. Some of this work concerns “don’t know” judgments of specific knowledge items and has been carried out in well confined task contexts in forensic research (e.g., Roebers et al., 2007; Buratti et al., 2014) and in memory studies (e.g., Glucksberg and McCloskey, 1981).

Knowledge assessments have also been investigated in research on *accuracy* of confidence judgments of one’s own and other people’s knowledge (e.g., Johansson and Allwood, 2007). Studies on confidence accuracy have in general shown that people often tend to be overconfident in their knowledge assessments although the reasons for this are contested (for reviews see Allwood and Granhag, 1999; Griffin and Brenner, 2004). In brief, this line of research shows the failure of both experts and novices to recognize their own ignorance in many contexts. Some research on confidence accuracy has also looked explicitly at expert-novice differences (e.g., Lichtenstein and Fischhoff, 1977; Lambert et al., 2012), however, the studies show mixed result and the differences found may depend on the knowledge domain.

Since studies regarding confidence accuracy for general knowledge concern evaluations of one’s own and others’ knowledge, they have some relevance to the present study. However, the results of the above mentioned studies usually pertain to confidence in answers to specific questions or confidence in memory of studied material. Thus, the knowledge assessments studied within the confidence accuracy literature differ from the broader knowledge assessments investigated in the present study. In addition, importantly, the accuracy of the performed knowledge assessments was not investigated in the present study.

Other research has studied the usefulness of perceived ignorance and has identified negative effects of knowledge (e.g., Son and Kornell, 2010; Fisher and Kail, 2016). For example, Son and Kornell (2010) reviewed research in cognitive psychology showing the advantages of *recognized* ignorance for individuals’ performance, for example with respect to the accuracy of their confidence judgments, judgments of learning, hindsight bias tendency, and learning (see also Smithson, 2015).

Within the fields of psychology of education and science communication studies research has investigated judgments concerning others’ knowledge (e.g., Scharrer et al., 2013, 2014; Shtulman, 2013; Bromme and Thomm, 2016; Bromme et al., 2016). Many such studies have investigated what is assumed to be known in research as well, but none of these studies have looked at the differences between experts and novices with respect to their knowledge assessments of all there is to know in disciplinary domains, which is the focus of the present study.

Finally, *ignorance*, in the sense described above, has been studied mainly in philosophy (e.g., Rescher, 2009) but also in risk research (e.g., Sjöberg, 2001). For example, several taxonomies of types of ignorance have been presented by philosophers and others (e.g., Smithson, 1989, 1993; Faber et al., 1992; Armour, 2000; Gross, 2007; Rescher, 2009; Croissant, 2014). In brief, although the above studies have investigated different types of judgments of perceived ignorance or knowledge, with the exceptions of risk research and philosophy, none of them have studied knowledge assessments of the broad kind examined in the present study.

Assessments of the extent of knowledge may be influenced by the judge’s conceptions of knowledge; that is, their epistemic beliefs which are part of their epistemic cognition (Sinatra et al., 2014). Such beliefs are both general and domain-specific (Hofer, 2006). Much research on epistemological beliefs, taking a general approach, has concluded that younger persons in many contexts may tend to view knowledge as something objective and absolute, where “right” or “wrong” exists. Later in development, the same research has identified a more subjective approach focused on the influence of “the knower” and knowledge, as a consequence, being seen as more uncertain. Further developments include more complicated epistemological beliefs where an objective and a subjective approach to knowledge are integrated at a more mature level. Here, knowledge is seen as more tentative and evolving and several different versions of the “truth” are possible (Perry, 1970; Kuhn et al., 2000; Sinatra et al., 2014). Kuhn et al. (2000) found that many of their adult participants had not reached the last developmental stage where the subjectivity of “the knower” is seen to mediate understanding of reality but where, at the same time, critical thinking can be an aid to enhance understanding of reality. Other approaches to epistemic cognition emphasize the context-bound and domain specific nature of epistemological thinking (e.g., Elby and Hammer, 2001).

In order to study how ignorance and knowledge are perceived we defined, on a general level, knowledge as *true*, *justified belief*, thereby following a common definition in Western philosophical tradition. True, justified belief means that, apart from truth, also both conviction and satisfactory reasons are necessary for the knowledge claim to be called knowledge. In this study, we, more specifically, identified as knowledge such conceptions about the world which at the present time are regarded as established according to accepted scientific standards. These conceptions, however, may be shown to be wrong in the future. With *ignorance* we mean lack of knowledge in a broad sense, that is, in relation to everything possible to know.

In this study we also tested a common idea, namely that the more you know, the more you know you don’t know. More specifically it has been argued that the more you know about an area, the more you realize you do not know (e.g., Ravetz, 1987; Krohn, 2001; Gross, 2007; Rescher, 2009; Gross and McGoey, 2015). For example, Gross (2007) suggested “whenever new knowledge arises the perceived amount of non-knowledge increases at least proportionally, since ‘every state of knowledge opens up even more notions of what is not known’ (Krohn, 2001, p. 8141)” (p. 751). Thus, it is argued that new knowledge gives a person more possibilities to identify new unknowns. Following this line of thinking, experts should be more aware of their ignorance than novices. However, we know of no empirical research pertaining to this conclusion.

The present study compared the knowledge assessments of experts and novices. In order to cover different scientific traditions, the four disciplines history, medicine, physics, and psychology were selected for the study. For example, psychology and physics may differ in that physics has a clearer consensus regarding its core content than psychology (Howell et al., 2014). However, the notion of expertise is somewhat vague. In expert research it has been argued that it takes about 10 years to become an expert in a field (Hayes, 1989; Ericsson, 1996), although the results in other studies have indicated that this time may be shorter for more gifted persons (Hambrick et al., 2014).

In a (somewhat insufficient) attempt to approximate this, experts in the present study were defined as persons with a Ph.D. degree and novices as 1st-year undergraduate students in the same discipline. Fisher and Kail (2016) differentiated between passive and formal expertise where passive expertise is acquired through every-day life experience and formal expertise through many years of study of some topic. The experts in the present study clearly belong to the formal type. Our expert participants can be assumed to use much of their expert knowledge actively in research and teaching and thereby to differ from the experts used by Fisher and Kail (2016) (defined as having completed a bachelor’s degree).

Out of the various dimensions of epistemic beliefs (see e.g., Schommer, 1990; Bråten and Strømsø, 2005; Teo, 2013; Sinatra et al., 2014) we, within the limited scope of this study, only investigated one dimension, *belief in certainty of knowledge*. Such belief has to do with “how tentative or absolute and stable knowledge is supposed to be” (Sinatra et al., 2014, p. 128; see also e.g., Bråten and Strømsø, 2005). In addition, we only looked at differences in general belief in certainty of knowledge and how it relates to the ignorance/knowledge assessments of experts and novices in different disciplines. Most research on epistemological beliefs has used students or adolescents as participants and the present study contributes by also investigating experts in different disciplines. Thus, by including persons with a Ph.D. degree the present study provides understanding of research disciplines as such, not just, for example, of students’ conceptions about the disciplines.

It is reasonable to think that experts, due to the expanded view into the unknown allowable from their greater platform of knowledge, should be able to see more of what is unknown with respect to both their own and their discipline’s knowledge. In order to test the idea that more knowledge is associated with more perceived ignorance (e.g., Gross, 2007) we formulated two hypotheses with respect to the relation between knowledge level (expert, novice) and experienced knowledge. Hypothesis 1 expected that the level of experts’ rated *own ignorance* would be *greater* (Study 1) and the level of *their own knowledge*, out of everything there is to know in their expert area, would be *lower* (Study 2), compared to novices’ ratings of the extent of their own knowledge. In line with this, Hypothesis 2 was that experts’ rated level of the extent of knowledge in their discipline out of everything there is to know in that discipline area would be *lower* than the corresponding rating by novices (Studies 1 and 2).

Our final two hypotheses related to the participants’ belief in certainty of knowledge. Hypothesis 3 was that experts would show a lower level of belief in certainty of knowledge than novices. This is in line with research on the development of epistemological beliefs (Perry, 1970; Kuhn et al., 2000; Sinatra et al., 2014). On the basis of this research it is reasonable to expect that the experts would show a higher level of epistemological development than the novices and thus lower belief in certainty of knowledge. Hypothesis 4 expected that the participants’ level of belief in certainty of knowledge would be positively associated with the level of their knowledge ratings for knowledge in their own and other disciplines. For people with a high level of belief in certainty of knowledge the task to establish knowledge is likely to seem simpler than for people with less belief in the certainty of knowledge. Given a higher level of belief in certainty of knowledge it would thus seem reasonable to be prepared to believe that more knowledge has been secured out of all possible knowledge to find. We did not expect that the participants’ ratings of *their own* ignorance/knowledge would be associated with their belief in certainty of knowledge, since assessments of one’s own knowledge may to a higher extent rely on other types of evidence than belief in certainty of knowledge.

## Study 1

Study 1 examined how experts and novices judged their ignorance when asked to think about what they *do not* know within a specific area and they were also asked *how much is known* in the same areas. In addition, ratings of belief in certainty of knowledge were studied.

### Method

#### Participants

The participants were 207 novices and 239 experts from nine Swedish universities. All participants were active in one of the four disciplines: history, medicine, physics, and psychology (see **Table 1**). The novices’ average age was 24 years (*SD* = 7.5) and 49% were women. The experts’ average age was 51 years (*SD* = 13.4) and 37% were women. The novices had on average 1 year (range 0–6, *SD* = 0.8) education experience within their discipline and the majority (94%) had no academic degree. The experts had an average of 22.2 years (range 1–70, *SD* = 13.8) research experience. Thirty-eight percent of the experts were research associates, 37% associate professors and 35% full professors.

**Table 1:** Distribution of participants in each discipline.

	Study 1			Study 2		
	Novices (*n*)	Experts (*n*)	Total (*N*)	Novices (*n*)	Experts (*n*)	Total (*N*)
History (*n*)	53	29	82	29	26	55
Medicine (*n*)	46	106	152	80	46	126
Physics (*n*)	64	48	112	42	25	67
Psychology (*n*)	44	56	100	43	44	87
Total (*N*)	207	239	446	194	141	335

#### Questionnaires

Data was collected using a web-based questionnaire. In the first part of the questionnaire the participants assessed their own ignorance, that is, how much knowledge they *do not* have of everything there is to know for a number of subareas in each of four disciplines. In the next part of the questionnaire, they assessed how much *is known* in a number of subareas in each of the four disciplines. Inspired by a study by Allwood and Granhag (1996) the two types of questions were (translated from Swedish) formulated as follows: “How much of everything there is to know in the following subject areas do you not have knowledge about?” and “How much of everything there is to know in the following subject areas is today known knowledge for one or several people?”. The participants were asked to make assessments in relation to “all there is to know” in each area with the explanation that “all there is to know” also includes such knowledge that is not known today, but which in the future may be. Both types of knowledge assessments were made on a scale where the response options ranged from 0% = *No knowledge*, to 100% = *All knowledge*, in increments of 10.

The four disciplines history, medicine, physics, and psychology were represented by 18 sub-disciplines (see Appendix A). Participants assessed both their own knowledge, and how much knowledge is known, for all 18 sub-disciplines in their own discipline and for 6 sub-disciplines within each of the other three disciplines (these sub-disciplines were the same for all participants; see Appendix A). In total, the survey consisted of 36 knowledge assessment items for each of the two types of knowledge assessments (own ignorance, knowledge in disciplines). The order of the different knowledge assessments as well as the sub-disciplines was counterbalanced with the exception that all participants judged their own discipline field first.

Finally, the web-questionnaire presented two items on belief in certainty of knowledge, taken from the “certainty of knowledge” factor, one of the four factors in the Bråten and Strømsø (2005) epistemological belief scale. The other three factors in the Bråten and Strømsø scale were labeled “speed of knowledge acquisition,” “knowledge construction and modification,” and “control of knowledge acquisition.” The two items used in the present study (“If scientists try hard enough, they can find the truth to almost everything” and “Scientists can ultimately get to the truth”) were the items with the highest factor loadings and the best face validity in the “certainty of knowledge” factor. The measure with these two items is henceforth called the Belief in Certainty of Knowledge Scale (BCKS). The items were rated on a scale that range from 1 = *Strongly disagree* to 5 = *Strongly agree*. A high value indicates a high belief in certainty of knowledge and a lower value indicates a more relativistic view.

#### Procedure

E-mail addresses were collected from the departments relating to history, medicine, physics, and psychology at Swedish universities. Experts and novices were contacted through their e-mail addresses with an invitation to participate in the web-based questionnaire. Those who participated were included in the draw for a gift certificate of 1,000 SEK (at the time approximately 115 USD). The questionnaire took about 10 min to answer. The invitation was sent to 2,216 persons and a reminder 1 week later. The initial response rate was 31.0%, more specifically, 31.6% of the experts and 30.6% of the novices started the survey. Data from participants who chose to cancel the form, or had more than five unanswered questions were excluded. The final response rate was 20.1%. More specifically, 18.7% of the experts, and 20.1% of the novices, completed the survey. According to chi-square analysis there was no difference between the number of experts and novices who started and completed the questionnaire (*p* > 0.05).

### Results

Means and standard deviations for the different knowledge assessments: own ignorance in own discipline, own discipline’s knowledge, own ignorance in the other three disciplines, and other disciplines’ knowledge, are presented separately for experts and novices in **Table 2**.

**Table 2:** Mean and standard deviations for the four types of knowledge assessments (in percentages), separately for experts and novices, in Studies 1 and 2.

	Study 1		Study 2	
	Experts	Novices	Experts	Novices
	*M* (*SD*)	*M* (*SD*)	*M* (*SD*)	*M* (*SD*)
Own ignorance – within own discipline	29.7 (19.6)	18.8 (17.7)	25.9 (18.1)	16.2 (15.5)
Knowledge – within own discipline	47.9 (21.9)	42.9 (22.2)	44.6 (21.5)	47.6 (21.8)
Own ignorance – within other disciplines	12.0 (18.0)	10.0 (13.5)	8.3 (9.7)	9.2 (10.3)
Knowledge – within other disciplines	48.5 (22.7)	47.1 (24.2)	46.0 (21.5)	51.3 (22.3)

To test the hypotheses we conducted analyses of variance. Theoretically a MANOVA should have been used to analyze the data, however, due to presence of multicollinearity between the dependent variables; this type of analyses was not performed. The dependent variables *own ignorance within own discipline*, *knowledge within own discipline*, *own ignorance within other disciplines*, *knowledge within other disciplines*, and *belief in certainty of knowledge* (BCKS) were therefore analyzed separately. More specifically, we performed five independent two-way ANOVA/ANCOVA’s with the between-group variables *knowledge level* (expert, novice) and *discipline* (history, medicine, physics, and psychology), and one mixed ANOVA with the within-group variable *knowledge assessment* (own discipline, other disciplines) and the between-group variable *discipline*. The BCKS ratings were included as a covariate when this measure was found to be correlated with the knowledge assessments. Hierarchical regression analysis was not used due to that the discipline variable does not have a clear reference group or logical order, which makes the interpretation of the correlation coefficients more difficult. Therefore, ANCOVA was better suited to analyze the effect of two categorical variables, and to control for the impact of a possible confounding variable, namely the BCKS. Finally, correlations between BCKS ratings and knowledge assessments were analyzed separately for experts and novices. Cronbach’s alpha for the participants’ assessments of their own ignorance was 0.96 (history: 0.97, medicine: 0.96, physics: 0.97, and psychology: 0.96), and 0.98 (history: 0.98, medicine: 0.98, physics: 0.98, and psychology: 0.98) for the assessments of how much that is known in the respective discipline. Cronbach’s alpha for the BCKS was 0.88.

#### Assessment of Own Ignorance in the Participants’ Own Discipline

In contrast to Hypothesis 1, experts (*M* = 29.7%, *SD* = 19.6%) estimated their own *ignorance* as *lower* than the novices (*M* = 18.8%, *SD* = 17.7%), *F*(1,437) = 42.04, *p* < 0.001, $\eta_{P}^{2}$ = 0.07. There was a main effect of discipline, *F*(3,437) = 9.82, *p* < 0.001. The LSD *post hoc* test showed that the historians (*M* = 33.0%, *SD* = 20.5%) estimated their own ignorance lower than the other disciplines (medicine: *M* = 23.7%, *SD* = 19.6%, physics: *M* = 19.5%, *SD* = 17.4%, psychology: *M* = 25.1%, *SD* = 18.6%), *p* < 0.01. No significant differences were found between medics, physicists, and psychologists, *p* > 0.05. There was no interaction effect between knowledge level and discipline, *F*(3,437) = 2.04, *p* = 0.108.

#### Assessments of Knowledge in the Participants’ Own Discipline

**FIGURE 1:**
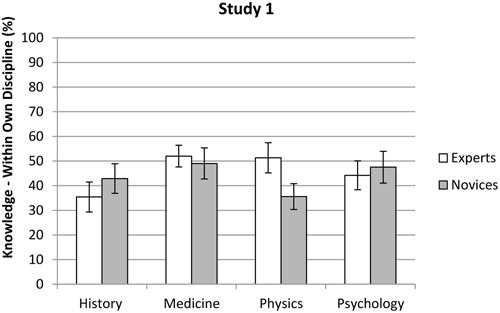
**Mean scores for experts and novices in the different disciplines with respect to their assessments of knowledge in their own discipline in Study 1.** Error bars indicate the 95% confidence interval.

In contrast to Hypothesis 2, no difference was found between experts’ (*M* = 47.9%, *SD* = 21.9%) and novices’ (*M* = 42.9%, *SD* = 22.2%) estimations of how much knowledge there is in their own discipline, *F*(1,404) = 1.78, *p* = 0.183. However, a main effect was found for discipline, *F*(3,404) = 4.12, *p* = 0.007, η^2^ = 0.03. The LSD *post hoc* test showed that medics (*M* = 51.0%, *SD* = 20.0%) estimated knowledge in their own discipline significantly *higher* than historians (*M* = 40.2%, *SD* = 23.9%) and physicists (*M* = 42.2%, *SD* = 23.4%), *p* < 0.01, but not significantly higher than psychologists (*M* = 45.7%, *SD* = 20.7%), *p* = 0.09. No significant differences were found between historians, physicists and psychologists, *p* > 0.05. An interaction effect was also found between knowledge level and discipline, *F*(3,404) = 4.74, *p* = 0.003, η^2^ = 0.03. Specifically, as shown in **Figure 1** (see also Appendix B), novices (*M* = 35.6%, *SD* = 20.6%) and experts (*M* = 51.3%, *SD* = 24.2%) in physics differed in their knowledge assessments, *p* < 0.001, while no differences were found for historians (novices: *M* = 42.9%, *SD* = 23.8%, experts: *M* = 35.4%, *SD* = 23.7%, *p* = 0.054), medics (novices: *M* = 49.0%, *SD* = 24.1%, experts: *M* = 52.0%, *SD* = 17.7%, *p* = 0.393), and psychologists (novices: *M* = 47.5%, *SD* = 17.8%, experts: *M* = 44.2%, *SD* = 22.9%, *p* = 0.373). The covariate, (i.e., the BCKS ratings) was related to the participants’ assessments of knowledge in their own discipline, *F*(1,404) = 10.25, *p* = 0.001, η^2^ = 0.02. Higher BCKS ratings were associated with higher knowledge assessments, *r* = 0.15, *p* < 0.01.

#### Assessment of Own Ignorance Outside the Participants’ Own Discipline

No significant main effect was found for knowledge level (experts, novices) when the participants rated their own ignorance outside their own discipline, *F*(1,427) = 0.96, *p* = 0.328, experts (*M* = 12.0%, *SD* = 18.0%) and novices (*M* = 10.0%, *SD* = 13.5%). Similarly, no main effect was found for discipline, *F*(3,427) = 0.25, *p* = 0.860. Thus, there were no significant differences between how historians (*M* = 10.6%, *SD* = 19.3%), medics (*M* = 11.5%, *SD* = 17.3%), physicists (*M* = 10.0%, *SD* = 12.8%), and psychologists (*M* = 12.0%, *SD* = 14.6%) rated their ignorance outside their own discipline. Moreover, there was no significant interaction effect between knowledge level and discipline, *F*(3,427) = 0.46, *p* = 0.709.

#### Assessments of Knowledge Outside the Participants’ Own Discipline

The results showed no main effect for knowledge level, *F*(1,394) = 0.02, *p* = 0.878. Thus, no difference was found between how experts (*M* = 48.5%, *SD* = 22.7%) and novices (*M* = 47.1%, *SD* = 24.2%) rated knowledge outside the own discipline. However, there was a main effect for discipline, *F*(3,394) = 8.19, *p* < 0.001, η^2^ = 0.06. The LSD *post hoc* test showed that historians (*M* = 35.2%, *SD* = 24.4%) estimated knowledge outside their own discipline *lower* than medics (*M* = 51.5%, *SD* = 21.1%), physicists (*M* = 51.9%, *SD* = 24.2%) and psychologists (*M* = 48.0%, *SD* = 21.7%), *p* < 0.01. No significant differences were found between medics, physicists, and psychologists, *p* > 0.05. No interaction effect was found between knowledge level and discipline, *F*(3,394) = 0.55, *p* = 0.652. The participants’ BCKS ratings were related to their assessments of knowledge outside their own discipline, *F*(1,394) = 7.44, *p* = 0.007, η^2^ = 0.02. Higher BCKS ratings were associated with higher knowledge assessments, *r* = 0.16, *p* < 0.01.

#### Comparison of Discipline-Related Knowledge Assessments between the Four Disciplines

**FIGURE 2:**
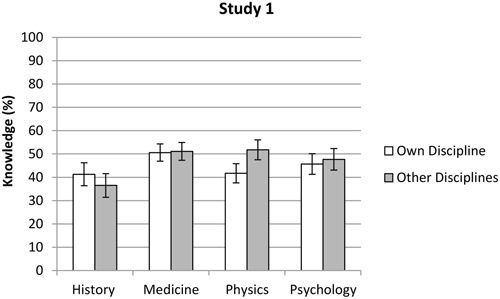
**Mean scores of the participants’ knowledge assessments within their own and other disciplines in Study 1.** Error bars indicate the 95% confidence interval.

There was a significant main effect for knowledge assessment, *F*(1,414) = 7.41, *p* = 0.007, η^2^ = 0.04. The participants’ knowledge assessments were significantly lower when they judged their own discipline (*M* = 45.4%, *SD* = 22.2%) compared with the other disciplines (*M* = 47.8%, *SD* = 23.3%). There was also a main effect for discipline, *F*(3,414) = 5.23, *p* = 0.001, η^2^ = 0.02. The LSD *post hoc* test showed that historians (*M* = 38.9%, *SD* = 24.8%) generally made lower knowledge estimations than the medics (*M* = 50.8%, *SD* = 20.5%), physicists (*M* = 46.8%, *SD* = 23.7%), and psychologists (*M* = 46.7%, *SD* = 21.0%), *p* < 0.05. No differences were found between the three other disciplines, *p* > 0.05. A significant interaction effect, *F*(3,414) = 17.80, *p* < 0.001, η^2^ = 0.13, showed that historians were the only group that rated knowledge in their own subject field (*M* = 41.3%, *SD* = 24.5%) higher than knowledge in other disciplines (*M* = 36.5%, *SD* = 25.1%). Hence, all the other groups estimated that there was more knowledge in the other three disciplines (medics: *M* = 51.1%, *SD* = 21.0%, physicists: *M* = 51.8%, *SD* = 24.1%, psychologists: *M* = 47.7%, *SD* = 21.4%), compared to their own area (medics: *M* = 50.6%, *SD* = 20.0%, physicists: *M* = 41.7%, *SD* = 23.3%, psychologists: *M* = 45.7%, *SD* = 20.6%, see **Figure 2** and Appendix B). Noteworthy, analysis of simple effects showed that the difference between the two knowledge assessments was significant only among the historians (*p* = 0.003) and the physicists (*p* < 0.001).

#### Comparison of Belief in Certainty of Knowledge between Novices and Experts and between Disciplines

There was a main effect of level of expertise *F*(1,411) = 8.14, *p* = 0.005, η^2^ = 0.02. In support of Hypothesis 3, novices (*M* = 2.7, *SD* = 1.2) believed more in certainty knowledge than experts (*M* = 2.5, *SD* = 1.3). A main effect of discipline was found, *F*(3,411) = 3.36, *p* = 0.019, η^2^ = 0.02. *Post hoc* analyses with Bonferroni corrections showed that the participants from the history discipline (*M* = 2.4, *SD* = 1.2) believed less in certainty knowledge than the other participants (physics: *M* = 2.8, *SD* = 1.2, psychology: *M* = 2.5, *SD* = 1.3, medicine: *M* = 2.6, *SD* = 1.2), *p* < 0.05. No significant interaction effect was found, *F*(3,411) = 2.25, *p* = 0.082, η^2^ = 0.02.

#### Relationship between Belief in Certainty of Knowledge and Knowledge Assessments

As can be seen in **Table 3**, the BCKS ratings were only significantly correlated with some knowledge assessments for the experts and none for the novices, thus providing partial support for Hypothesis 4. For experts, the BCKS ratings were significantly correlated with knowledge assessments involving the discipline’s knowledge [within own discipline: *r*(212) = 0.28, *p* < 0.001, within other disciplines: *r*(204) = 0.28, *p* < 0.001], but not the participant’s own knowledge [within own discipline: *r*(217) = 0.07, *p* = 0.298, within other disciplines: *r*(215) = 0.02, *p* = 0.731]. For novices, no significant correlations were found between the BCKS ratings and assessments of the participants’ own knowledge [within own discipline: *r*(197) = 0.10, *p* = 0.171, within other disciplines: *r*(197) = 0.08, *p* = 0.294] or the discipline’s knowledge [within own discipline: *r*(197) = 0.04, *p* = 0.542, within other disciplines: *r*(195) = 0.04, *p* = 0.583].

**Table 3:** Bivariate correlations between belief in certainty of knowledge and the four types of knowledge assessments.

	Belief in certainty of knowledge			
	Study 1		Study 2	
	Experts	Novices	Experts	Novices
Own ignorance – within own discipline	0.07	0.10	0.07	0.09
Knowledge – within own discipline	0.28***	0.04	0.22*	0.18*
Own ignorance – within other disciplines	0.02	0.08	0.12	0.03
Knowledge – within other disciplines	0.28***	0.04	0.29**	0.14

*p < 0.05, **p < 0.01, ***p < 0.001.

## Study 2

In order to explore if ratings of ignorance differ from ratings of knowledge, we conducted a second study, replicating Study 1 with the exception that the participants in Study 2 were asked to rate their *knowledge* instead of their *ignorance* (as in Study 1). A similar framing manipulation has previously been used in a study by Allwood and Granhag (1996), where they concluded that the knowledge assessments were unaffected by this manipulation. Therefore, in line with these findings, we expected Study 2 to replicate the results from Study 1.

### Method

#### Participants

The participants were 194 novices and 141 experts from 17 Swedish universities. As in Study 1, the recruited participants were active in one of the four disciplines history, medicine, physics, or psychology (see **Table 1**). The novices’ average age was 25 years (*SD* = 7.1) and 56% were women. The experts’ average age was 51 years (*SD* = 10.9) and 39% were women. The novices had on average 1 year (range 0–6, *SD* = 0.8) education experience within their discipline and the majority (94%) had no academic degree. The experts had an average of 18.9 years (range 2–80, *SD* = 11.4) research experience. Forty-eight percent were research associates, 28% associate professors and 24% full professors.

#### Questionnaires

Study 2 was conducted with the same questionnaire as Study 1, with the exception that the framing was changed so that the participants now judged how much knowledge *they have*, instead of how much *they do not have* (“How much of everything there is to know in the following subject areas do you have knowledge about?”). The response scale was the same in both studies.

#### Procedure

Study 2 was conducted in the same way as Study 1. Individuals who had participated in Study 1 were excluded. The invitation, with reminder, was sent to 1,635 individuals. The initial response rate was 22.3%. 25.9% of the experts and 20.2% of the novices started the survey. Just as in Study 1, we excluded participants who decided to cancel the questionnaire or had more than five unanswered questions. The final response rate was 20.5%; 24.0% of the experts and 18.5% of the novices completed the survey. Chi-square analysis showed that more experts started and completed the questionnaire than novices (*p* < 0.01).

### Results

Means and standard deviations for the different knowledge assessments of: own knowledge in own discipline, own discipline’s knowledge, own knowledge in the three other disciplines, and other disciplines’ knowledge, are presented separately for experts and novices in **Table 2**. Just as in Study 1, the hypotheses were investigated with five independent two-way ANOVA/ANCOVA’s with the between-group variables *knowledge level* (expert, novice) and *discipline* (history, medicine, physics, and psychology), and one mixed ANOVA with the within-group variable *knowledge assessment* (own discipline, other disciplines) and the between-group variable *discipline*. BCKS was included as a covariate when it was found to be significantly correlated with the knowledge assessments. Correlations between BCKS ratings and knowledge assessments were analyzed separately for experts and novices. Cronbach’s alpha for the participants’ assessments of their own knowledge was 0.94 (history: 0.97, medicine: 0.96, physics: 0.96, and psychology: 0.97) and for their assessments of how much that is known in the respective disciplines, 0.98 (history: 0.99, medicine: 0.98, physics: 0.98, and psychology: 0.98). The BCKS showed a Cronbach’s alpha of 0.84.

#### Assessments of Own Knowledge in the Participants’ Own Discipline

In contrast to our first hypothesis, experts (*M* = 25.9%, *SD* = 18.1%) estimated their own knowledge *higher* than the novices (*M* = 16.2%, *SD* = 15.5%), *F*(1,312) = 17.48, *p* < 0.001, η^2^ = 0.05. The results also showed a main effect for discipline, *F*(3,312) = 15.59, *p* < 0.001, η^2^ = 0.12. The Games-Howell *post hoc* test showed that historians (*M* = 26.3%, *SD* = 17.1%) and psychologists (*M* = 29.2%, *SD* = 19.8%) rated their knowledge significantly *higher* than both physicists (*M* = 13.1%, *SD* = 13.0%) and medics (*M* = 15.6%, *SD* = 14.0%), *p* < 0.001. There was no significant interaction effect between knowledge level and discipline, *F*(3,312) = 2.27, *p* = 0.080.

#### Assessments of Knowledge in the Participants’ Own Discipline

In contrast to our second hypothesis, no significant difference was found between experts (*M* = 44.6%, *SD* = 21.5%) and novices (*M* = 47.6%, *SD* = 21.8%) when they estimated how much knowledge that is known in their own discipline, *F*(1,285) = 0.93, *p* = 0.335. However, a significant main effect was found for discipline, *F*(3,285) = 4.17, *p* = 0.007, η^2^ = 0.04. The LSD *post hoc* test showed that historians (*M* = 39.7%, *SD* = 22.6%) and physicists (*M* = 41.4%, *SD* = 22.3%) rated the knowledge in their own discipline as lower than both medics (*M* = 49.6%, *SD* = 21.7%) and psychologists (*M* = 49.8%, *SD* = 19.3%), *p* < 0.05. No interaction effect was found between knowledge level and discipline, *F*(3,285) = 1.25, *p* = 0.292. The participants’ BCKS ratings were related to their assessments of knowledge in their own discipline, *F*(1,285) = 10.38, *p* = 0.001, η^2^ = 0.03. Higher ratings were associated with higher knowledge assessments, *r* = 0.21, *p* < 0.01.

#### Assessment of Own Knowledge Outside the Participants’ Own Discipline

**FIGURE 3:**
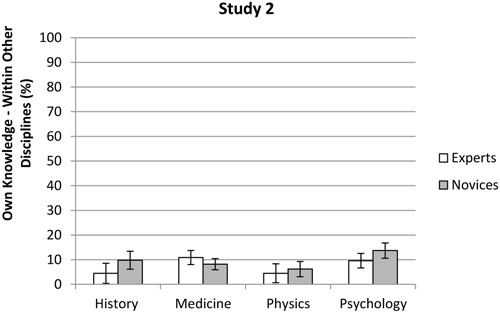
**Mean scores for experts and novices in the different disciplines with respect to their assessments of their own knowledge outside their own discipline in Study 2.** Error bars indicate the 95% confidence interval.

No significant difference was found between experts’ (*M* = 8.3%, *SD* = 9.7%) and novices’ (*M* = 9.2%, *SD* = 10.3%) ratings of their own knowledge outside their own discipline, *F*(1,305) = 3.20, *p* = 0.075. However, there was a main effect for discipline, *F*(3,305) = 5.58, *p* = 0.001, η^2^ = 0.05. The Games-Howell *post hoc* test showed that physicists (*M* = 5.5%, *SD* = 4.9%) rated their knowledge outside their own discipline significantly *lower* than both medics (*M* = 9.2%, *SD* = 10.1%), and psychologists (*M* = 11.6%, *SD* = 12.0%), *p* < 0.05, but not significantly lower than the historians (*M* = 7.5%, *SD* = 10.0%), *p* > 0.05. There was also an interaction effect between knowledge level and discipline, *F*(3,305) = 2.89, *p* = 0.036, η^2^ = 0.03. Within medicine, the experts (*M* = 10.9%, *SD* = 11.3%) rated their knowledge higher than the novices (*M* = 8.2%, *SD* = 9.3%, see **Figure 3** and Appendix B). Within the three other disciplines the novices (history: *M* = 9.8%, *SD* = 12.2%, physics: *M* = 6.2%, *SD* = 5.1%, psychology: *M* = 13.7%, *SD* = 12.9%) gave higher knowledge assessments than the experts (history: *M* = 4.5%, *SD* = 4.9%, physics: *M* = 4.5%, *SD* = 4.5%, psychology: *M* = 9.6%, *SD* = 10.8%). However, analyses of simple effects showed no significant differences between the experts and the novices within each discipline, *p* > 0.05.

#### Assessments of Knowledge Outside the Participants’ Own Discipline

No difference was found between how experts (*M* = 46.0%, *SD* = 21.5%) and novices (*M* = 51.3%, *SD* = 22.3%) rated knowledge outside their own discipline, *F*(1,278) = 2.84, *p* = 0.093. However, a main effect was found for discipline, *F*(3,278) = 5.03, *p* = 0.002, η^2^ = 0.05. The Bonferroni *post hoc* test showed that historians (*M* = 36.6%, *SD* = 24.0%) estimated knowledge outside their own discipline significantly *lower* than both medics (*M* = 51.1%, *SD* = 22.6%), physicists (*M* = 50.6%, *SD* = 21.4%), and psychologists (*M* = 52.3%, *SD* = 18.6%), *p* < 0.05. However, no significant differences were found between medics, physicists, and psychologists, *p* > 0.05. No significant interaction effect was found between knowledge level and discipline, *F*(3,278) = 0.25, *p* = 0.865. Furthermore, a relationship was found between the participants’ BCKS ratings and the assessments of knowledge outside their own discipline, *F*(1,278) = 8.60, *p* = 0.004, η^2^ = 0.03. Higher ratings on the scale were associated with higher knowledge assessments, *r* = 0.22, *p* < 0.01.

#### Comparison of Discipline-Related Knowledge Assessments between the Four Disciplines

**FIGURE 4:**
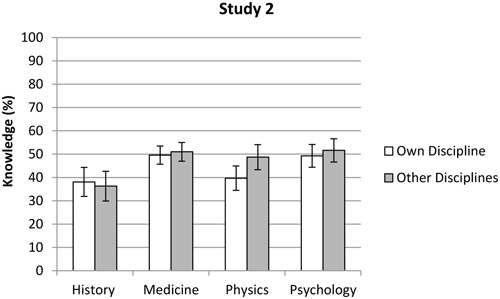
**Mean scores of the participants’ knowledge assessments within their own and other disciplines in Study 2.** Error bars indicate the 95% confidence interval.

The results showed a significant main effect for knowledge assessment, *F*(1,297) = 12.47, *p* < 0.001, η^2^ = 0.07. The participants’ knowledge assessments were significantly lower when they were judging their own discipline (*M* = 45.6%, *SD* = 21.8%) compared with the other disciplines (*M* = 48.4%, *SD* = 22.5%). There was also a main effect for discipline, *F*(3,297) = 5.51, *p* = 0.001, η^2^ = 0.04. The LSD *post hoc* test showed that historians (*M* = 37.2%, *SD* = 22.7%) generally made lower knowledge estimations than both the medics (*M* = 50.3%, *SD* = 21.8%) and the psychologists (*M* = 50.5%, *SD* = 19.4%), *p* < 0.01. However, no significant differences were found between medics, psychologists, and physicists (*M* = 44.2%, *SD* = 22.9%), *p* > 0.05. A significant interaction effect, *F*(3,297) = 7.65, *p* < 0.001, η^2^ = 0.08, showed that historians were the only group that rated knowledge in their own subject field (*M* = 38.1%, *SD* = 21.7%) higher than knowledge in other disciplines (*M* = 36.3%, *SD* = 23.7%). Hence, all the other groups estimated that there was more knowledge in the other three disciplines (medics: *M* = 51.0%, *SD* = 22.2%, physicists: *M* = 48.7%, *SD* = 22.9%, psychologists: *M* = 51.6%, *SD* = 19.4%), compared to their own area (medics: *M* = 49.6%, *SD* = 21.4%, physicists: *M* = 39.7%, *SD* = 22.9%, psychologists: *M* = 49.3%, *SD* = 19.5%, see **Figure 4** and Appendix B). Noteworthy, analysis of simple effects showed that the difference between the two knowledge assessments was significant only among the physicists (*p* < 0.001).

#### Comparison of Belief in Certainty of Knowledge between Novices and Experts and between Disciplines

There was a main effect of level of expertise *F*(1,296) = 10.36, *p* = 0.001, η^2^ = 0.03. In support of our Hypothesis 3, novices (*M* = 2.9, *SD* = 1.1) believed more in certainty of knowledge than experts (*M* = 2.5, *SD* = 1.2). A main effect of discipline was also found, *F*(3,296) = 3.18, *p* = 0.024, η^2^ = 0.03. *Post hoc* analyses with LSD adjustments showed that the historians (*M* = 2.3, *SD* = 1.2) believed less in certainty knowledge than the medics (*M* = 2.8, *SD* = 1.1) and physicists (*M* = 3.0, *SD* = 1.2), *p* < 0.05. No significant differences were found between medics, physicists, and psychologists (*M* = 2.6, *SD* = 1.1), *p* > 0.05. No significant interaction effect was found, *F*(3,296) = 1.81, *p* = 0.145, η^2^ = 0.02.

#### Relationship between Belief in Certainty of Knowledge and Knowledge Assessments

With partial support for Hypothesis 4, the BCKS ratings were significantly correlated with the experts’ knowledge assessments that involved the discipline’s knowledge [within own discipline: *r*(119) = 0.22, *p* = 0.013, within other disciplines: *r*(115) = 0.29, *p* = 0.002], but not with assessments of one’s own knowledge [within own discipline: *r*(126) = 0.07, *p* = 0.455, within other disciplines: *r*(123) = 0.12, *p* = 0.200], see **Table 3**. For the novices, the BCKS ratings were found to be significantly correlated with assessments of the known knowledge in the own discipline [*r*(171) = 0.18, *p* = 0.017] but not within other disciplines [*r*(168) = 0.14, *p* = 0.080], and not with assessments of own knowledge [within own discipline: *r*(172) = 0.09, *p* = 0.237, within other disciplines: *r*(172) = 0.03, *p* = 0.704].

## General Discussion

The present study investigated experts’ and novices’ assessments of their own knowledge and of the extent of knowledge in general within their own and other disciplines out of everything there is to know. In order to study potential disciplinary differences we included participants from history, medicine, physics, and psychology. We also asked the participants about their general belief in certainty of knowledge in order to examine the relation between this belief and the level of the participants’ knowledge assessments.

We first tested if the commonly known assertion “the more you know, the more you realize you do not know” is correct. If so, experts in an area should rate themselves as more ignorant and less knowledgeable in that area compared to novices (Hypothesis 1). In contrast to our expectation, the results, over both studies, showed that the experts rated their own knowledge higher than the novices rated their own knowledge, irrespective of whether they assessed their ignorance or their knowledge. Although this result refuted our hypothesis, it is in line with the expected actual knowledge level of experts and novices. The knowledge assessments in this study can be seen as *global judgments* involving a smaller or larger knowledge area. This type of judgment has in research on confidence judgments been found to be associated with a broader range of considerations by the person making the judgment, compared with *item specific judgments* where the person considers matters more specific to the item (Sniezek and Buckley, 1991). The experts’ self-understanding as experts, in the sense of being knowledgeable about the area (and thus as persons who are likely to know a lot), may have contributed to their higher knowledge assessments. Following this reasoning, the same considerations may have led novices to decrease their knowledge ratings.

We also expected experts to rate what is known within their own discipline (out of all there is to know) lower compared to the novices (Hypothesis 2). Studies 1 and 2 showed no support for this hypothesis. Overall, with the exception of physics in Study 1 (where the experts rated the knowledge as greater), experts and novices did not differ with respect to their perception of how much is known in their discipline. Given the lack of support for Hypotheses 1 and 2, “one of the most pervasive conceptual metaphors,” that is, the perception metaphor “knowing is seeing” (Johnson, 1993, p. 419), and specifically thinking about this seeing as seeing over fields or areas of knowledge, may not be fully appropriate. Instead, other ways of thinking about ignorance might be more fruitful to use, for example walking in a thick jungle where it is difficult to see the environment.

The only result remotely supporting Hypotheses 1 and 2 was found when the assessments of knowledge within one’s own discipline were compared with the same persons’ assessments of knowledge in other disciplines. Here the results showed that, especially in physics, the perceived knowledge in one’s own discipline was less than what was perceived in other disciplines. However, this tendency was small in psychology and medicine and was reversed in history. Thus, it seems fair to conclude that this result is apparent only for physics. Nevertheless, this finding is interesting in its own right, considering reports that at least some researchers in physics at the end of the 19th century thought that most of what there is to know on a general level in physics was known. For example, in a dedication speech for Ryerson Physics Lab, at the University of Chicago in 1894, Albert Michelson is reputed to have stated that “the more important fundamental laws and facts of physical science have all been discovered, and these are so firmly established that the possibility of their ever being supplanted in consequence of new discoveries is exceedingly remote [...] Our future discoveries must be looked for in the sixth place of decimals” (cited in Mari, 2005, p. 260). Given that our results generalize to other samples, physicists’ perception of how much remains to be known in their discipline has changed a lot since Michelson’s time.

In this context it is also worth to note that, in line with our expectation, the framing of the assessment question in terms of ignorance (Study 1) or knowledge (Study 2) did not influence the results very much since Study 2 to a large extent replicated the results of Study 1. This result is in line with previous research by Allwood and Granhag (1996) where two different samples were used and the framing of the knowledge assessment, as relating to the participants’ ignorance or knowledge, did not appear to influence the assessments. This result suggests that people may use similar cues when assessing their ignorance in a domain as when assessing their knowledge, perhaps influenced by examples of knowledge on both occasions. It is possible that humans are more naturally oriented toward thinking about what they know compared with thinking about what they do not know, just as people are more willing to seek confirmatory than disconfirmatory feedback regarding their hypotheses (confirmation bias, e.g., Nickerson, 1998; Jonas et al., 2001). This may be one reason why the framing used in Study 1 (“ignorance”) and Study 2 (“knowledge”) did not make much difference for the participants’ knowledge assessments. Future research should continue to examine factors that may contribute to an awareness of our ignorance. Such understanding could, for example, help prevent erroneous risk assessments (Ravetz, 1987, 1993).

It is also of interest to consider the absolute level of the assessments. Here it is noteworthy that the knowledge assessments were, in general, quite high. For instance, the participants estimated that about 40–50% *of all there is to know* within their own discipline is already known. Furthermore, the average difference between the experts and novices was quite small. In our two studies, the experts estimated that *they themselves* knew approximately 26–30% of all there is to know in their discipline and the corresponding rating for novices was 16–19%. It is also of relevance to note that the only identified difference between experts and novices was when they assessed their own knowledge within their own discipline.

### Differences in the Knowledge Assessments between the Disciplines

Although the results show that the differences between experts and novices were largely unaffected by discipline, our study contributes to understanding differences between the four disciplines. For instance, assessments from participants within the history discipline tended to stand out from those of the other disciplines. In both studies, historians (including both experts and novices) reported that they themselves knew more of their discipline compared to the corresponding ratings by members of the other disciplines. Moreover, in both studies, historians and physicists assessed that less was known within their discipline (about 40% of all there is to know) compared to medics (approximately 50%). In addition, compared to the participants in the three other disciplines, only the historians rated the level of knowledge in other disciplines as lower than that in their own discipline. In other words, historians appeared to be less impressed with the extent of disciplinary knowledge than members of the other disciplines. These findings are in line with research showing that historians have a greater awareness of the relativity, but still usefulness, of different perspectives in order to better understand historical events (Wineburg, 1998; Wolfe and Goldman, 2005). We suggest that future research, aiming to improve our understanding of research disciplines’ self-understanding and their understanding of other disciplines, would benefit from further exploring researchers’ assessments of the extent of disciplines’ knowledge and the limitations of such knowledge.

### Belief in Certainty of Knowledge

Studies 1 and 2 also showed consistency in the ratings of BCKS for the two levels of expertise, with respect to different disciplines, and the relationship between BCKS and knowledge assessments. In line with previous developmental research on belief in certainty of knowledge (e.g., Kuhn et al., 2000) and Hypothesis 3, the experts reported a lower belief in certainty of knowledge than the novices. It can be noted that the influence of experts’ lower degree of belief in certainty of knowledge did not stop them from assessing the level of their knowledge as greater than that of the novices.

Hypothesis 4 expected that the BCKS ratings would be positively associated with the assessments of knowledge in one’s own and the other disciplines. This hypothesis received partial support. The expected relationship was found for assessments of known knowledge within disciplines among experts, but, in general, not among novices. Moreover, in line with our hypothesis, the BCKS ratings were not correlated with the assessments of one’s own knowledge. These findings suggest that the understanding of *own knowledge* within and outside one’s own discipline, and for experts as well as novices, may be influenced by other types of cues that are more relevant for the assessments of own knowledge, for example their awareness of being an “expert” or “novice” within the area. However, only the experts’ knowledge may have been sufficiently well-integrated to be affected by their general belief in certainty of knowledge. Future research should continue to explore these issues, for example with respect to the relation between age and use of epistemic beliefs when assessing the extent of knowledge in different knowledge domains such as research disciplines.

The results also showed that the disciplines differed in degree of belief in certainty of knowledge. Thus, the historians showed a lower level of belief in certainty of knowledge than participants from the other disciplines. This finding may contribute to explain why historians, in general, made lower knowledge assessments than participants from other disciplines. However, the design used in the present study does not allow for causal inferences about how belief in certainty of knowledge affects knowledge assessments. It is possible that knowledge assessments as well as belief in certainty of knowledge are influenced by a general epistemological ethos. Future research should seek to explore this issue further.

Our results are of interest in different practical contexts such as higher education teaching contexts. For example, it might be relevant for teachers in the respective disciplines to note that the difference in the experts’ (26–30%) and novices’ (16–19%) estimates of how much they know of all there is to know in their discipline can be seen as fairly low. This could indicate that at least the novices’ (students’) estimates are inflated and that they might be more open to the challenges of research if they had somewhat more modest assessments of their own knowledge. Furthermore, our results concerning differences between experts and novices in degree of certainty of knowledge suggest that students may profit by more explicit teaching in epistemological aspects of their discipline. Moreover, our results are also of interest in interdisciplinary collaboration contexts. In such contexts a commonly recognized difficulty is that researchers have insufficient understanding of the characteristics of research in other disciplines than their own and our results can contribute to such understanding (Defila and Di Giullo, 2010).

### Limitations

The negation used in the framing of Study 1, where the participants were asked to rate what they do not know, may have caused confusion for some participants. This issue was dealt with in Study 2, where the negation was removed and the results, to a great extent, were replicated. This is in line with research by Allwood and Granhag (1996) showing that knowledge assessments may not be sensitive to these types of framing effects (i.e., ratings of *knowledge* or *ignorance*). Thus, this problem may not have affected the results to any greater extent.

As expected, strong correlations were found between level of expertise (expert, novice) and age (Study 1, *r* = 0.77; Study 2, *r* = 0.83). Although our experts and novices were clearly different in terms of disciplinary experience (education, research experience) and in terms of academic job experience (presumably also including teaching the discipline), because of the just mentioned strong correlations the difference between experts and novices could at least partly be conceived to be an effect of age rather than of knowledge. The fact that we found no difference between experts’ and novices’ knowledge assessments outside their own discipline indicates that such an effect of age at least probably was not absolute. If age had explained the results rather than expertise one would have expected an expert-novice difference also outside the experts’ own discipline, even if smaller than the difference within the own discipline. However, it is still possible that age to some degree may also have contributed to the expert-novice differences found. In general, it is not unproblematic to separate these factors because excellence requires many years of practical and theoretical experience (Ericsson et al., 2006). Likewise, a general, and possibly relevant, effect of age may be “cognitive maturation,” but such maturation is also likely to be influenced by the difference in academic training between the experts and novices. Future studies should examine if age correlates with greater insight into one’s own ignorance, regardless of the individual’s knowledge.

It should also be noted that there was a high internal consistency in the knowledge estimates. The fact that individuals are likely to make similar assessments across different disciplines indicates that the assessments were to some extent driven by general personal tendencies. Notably, the internal consistency were slightly higher for estimates of known knowledge compared to own knowledge, which indicates that individuals are more likely to differentiate between disciplines in assessments concerning their own knowledge. In this context it is of interest that the participants’ shown insensitivity to the differences between the disciplines with respect to known knowledge matches the finding in research on confidence judgments of a general confidence trait in adults (Kleitman and Stankov, 2007). Despite this, we found differences also for assessments of known knowledge; the participants rated knowledge in their own discipline lower compared to disciplines outside the participants’ own subject area.

A further limitation is the low response rates in both studies (approximately 20%). The samples could therefore be unrepresentative for their populations. This is a common issue with this type of data collection. However, the fact that many of the results were replicated in two studies conducted with independent samples should be considered as a strength in this context.

Future studies should investigate if our results hold for experts and novices from other domains. It is possible that experts within other, and non-academic, domains would consider knowledge as more fixed and stable and therefore rate their own as well as known knowledge higher than academic experts.

## Ethics Statement

The present research does not fall under the area of relevance of the Swedish ethical review. However, its ethical aspects and considerations were approved by the head of the Department of Psychology, University of Gothenburg. The research was carried out in accordance with guidelines of the the Swedish Research Council. Participants in the study granted their informed consent.

## Author Contributions

IH was responsible for data collection and statistical analyses. IH, SB, and CMA contributed equally in the development of study design and preparation of manuscript.

## Conflict of Interest Statement

The authors declare that the research was conducted in the absence of any commercial or financial relationships that could be construed as a potential conflict of interest.
