# Supplementary material for: Experts’ and Novices’ Perception of Ignorance and Knowledge in Different Research Disciplines and Its Relation to Belief in Certainty of Knowledge
Source: Front Psychol. 2017 Mar 17;8:377. doi: 10.3389/fpsyg.2017.00377 (PMC5355420; doi:10.3389/fpsyg.2017.00377)
Supplement: Supplementary file 1 [file Data_Sheet_1.docx]

**Appendix A**

The disciplines and sub-disciplines used in Study 1 and 2. Each participant assessed both their own ignorance and how much knowledge that is known in each subject area, for all eighteen disciplines in their own discipline and six (marked by *) in each of the others.

| **Psychology** | **Medicine** | **History** | **Physics** |
| --- | --- | --- | --- |
| Biological psychology | Anatomy* | Economic history* | Quantum physics |
| Cognitive psychology* | Physiology | Cultural history | Nuclear physics |
| Personality psychology* | Surgery* | History of ideas | Particle physics |
| Social psychology* | Genetics | Naval history | Atomic physics |
| Addiction psychology* | Pathology | Military history* | Molecular physics |
| Forensic psychology* | Neurology | Political history | Plasma physics* |
| Developmental psychology | Odontology* | History of religions* | Space physics |
| Neuro psychology | Dermatology | Social history | Astrophysics |
| Perceptual psychology | Pharmacology* | Women’s history | Biophysics |
| Economic psychology | Anesthesiology | History of Sweden | Geophysics* |
| Parapsychology | Internal medicine | Art history | Medical physics* |
| Psychology of emotions | Embryology* | History of Europe* | Applied physics* |
| Psychology of motivation | Urology | History of Africa* | Experimental physics* |
| Psychology of learning | Epidemiology | History of North America | Nanophysics |
| Organizational psychology* | Histology | History of South America | Condensed matter physics |
| Health psychology | Immunology | History of Asia* | Statistical physics |
| Evolutionary psychology | Microbiology | History of Oceania | Theoretical/mathematical physics* |
| Psychology of religion | Psychiatry* | History of Antarctica | Materials physics |
| *Note*. *Subject areas assessed by all participants. | | | |

**Appendix B**

Complementing figures showing the distributions of the knowledge ratings.


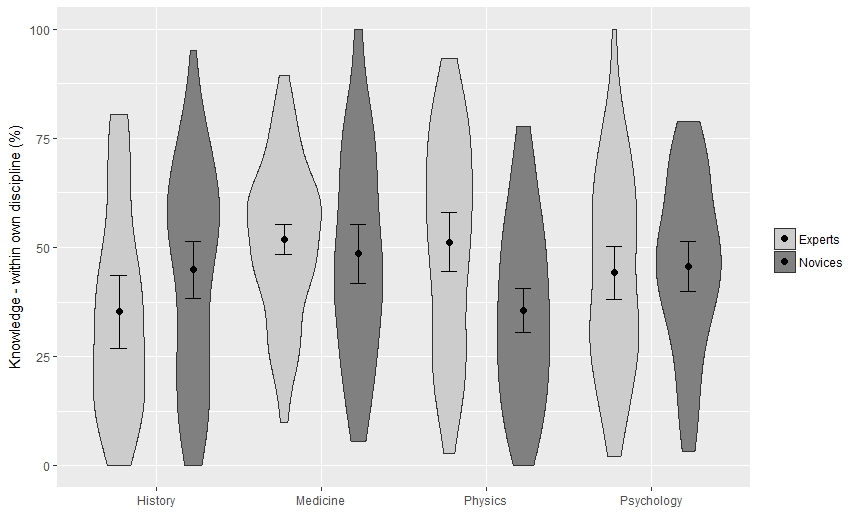


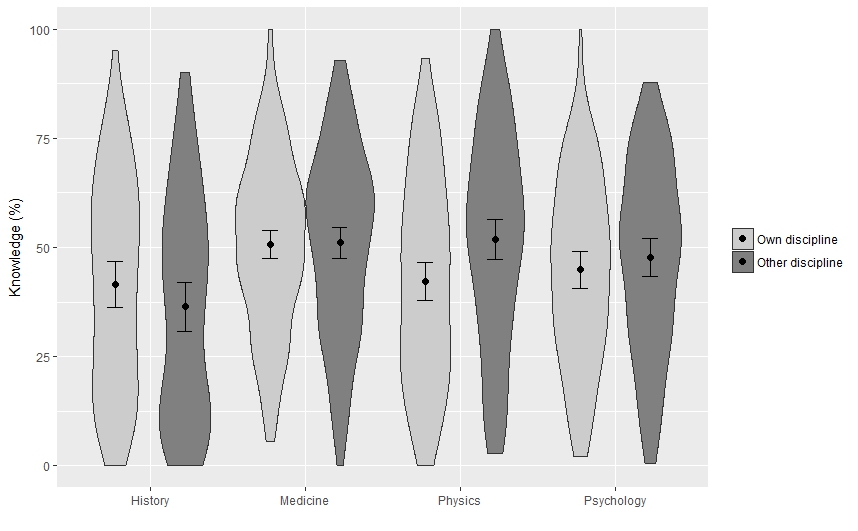
*Figure 1B.* Violin plot showing the distributions and the mean scores for experts and novices in the different disciplines with respect to their assessments of knowledge in their own discipline in Study 1. Error bars indicate the 95 % confidence interval.

*Figure 2B.* Violin plot showing the distributions and the mean scores of the participants’ knowledge assessments within their own and other disciplines in Study 1. Error bars indicate the 95 % confidence interval.


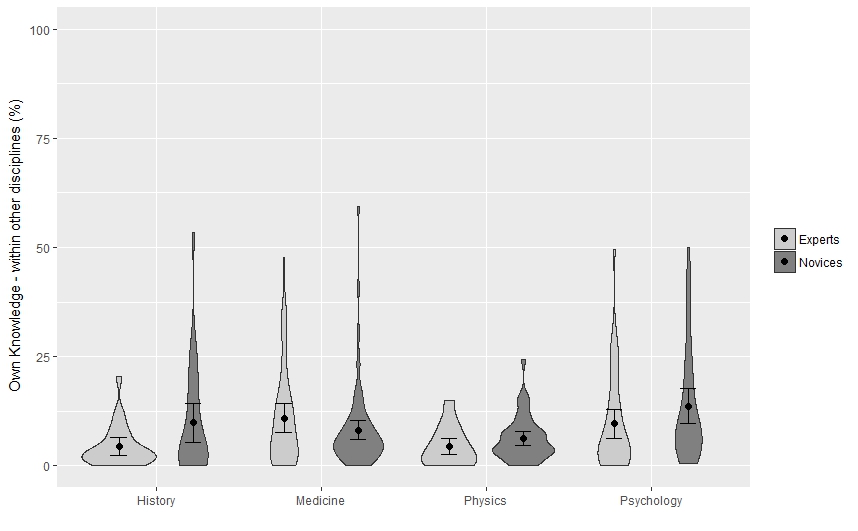


*Figure 3B.* Violin plot showing the distributions and the mean scores for experts and novices in the different disciplines with respect to their assessments of their own knowledge outside their own discipline in Study 2. Error bars indicate the 95 % confidence interval.


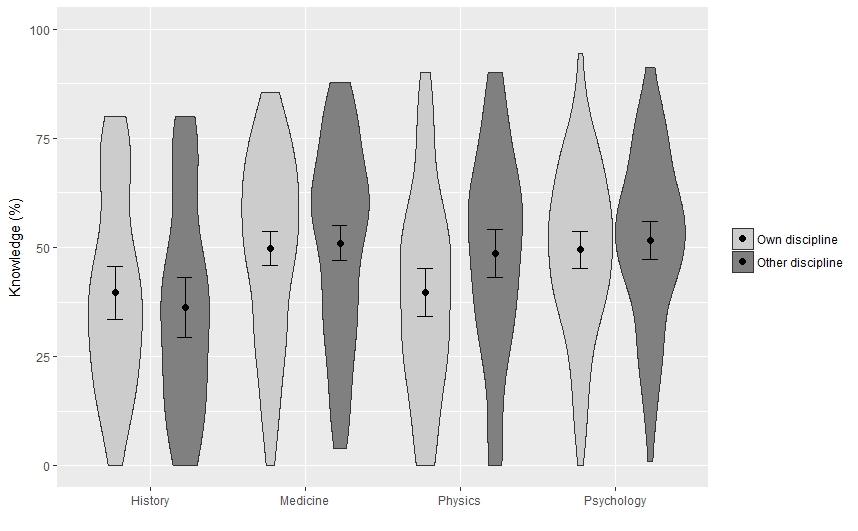


*Figure 4B.* Violin plot showing the distributions and the mean scores of the participants’ knowledge assesments within their own and other disciplines in Study 2. Error bars indicate the 95 % confidence interval.
